# Supplementary material for: Phosphate Limitation Modulates Vibrio Cholerae Outer Membrane Vesicle Formation, Composition and Toxicity
Source: J Extracell Biol. 2026 May 9;5(5):e70138. doi: 10.1002/jex2.70138 (PMC13157583; doi:10.1002/jex2.70138)
Supplement: Supplementary file 1 — Supplementary Figure S1. Particle‐to‐protein Ratio of OMV Preparations From Vibrio Cholerae N16961 and ΔphoB Strains Grown Under High and Low Phosphate Conditions [file JEX2-5-e70138-s002.pdf]

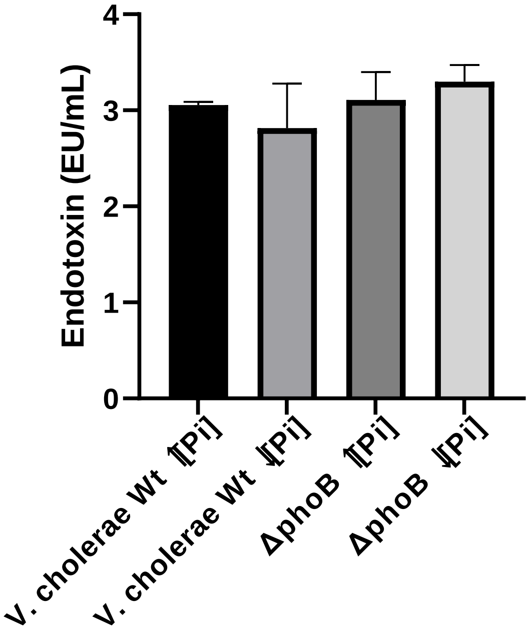

Supplementary Figure S2. Quantification of LPS in OMV preparations by LAL assay.Endotoxin levels were measured in OMV preparations obtained from *Vibrio cholerae* N16961 and *ΔphoB* strains grown under high- and low-phosphate conditions using the Limulus amebocyte lysate (LAL) assay. Data are presented as mean ± SD from three independent biological replicates. Statistical analysis was performed using ordinary one-way ANOVA followed by Tukey’s multiple-comparisons test. No significant differences were detected among OMV preparations
