# Supplementary material for: Phosphate Limitation Modulates Vibrio Cholerae Outer Membrane Vesicle Formation, Composition and Toxicity
Source: J Extracell Biol. 2026 May 9;5(5):e70138. doi: 10.1002/jex2.70138 (PMC13157583; doi:10.1002/jex2.70138)
Supplement: Supplementary file 2 — Supplementary Figure S2. Quantification of LPS in OMV Preparations by LAL Assay. S1 Table. Identified Proteins in OMVs by LC‐MS/MS [file JEX2-5-e70138-s001.pdf]

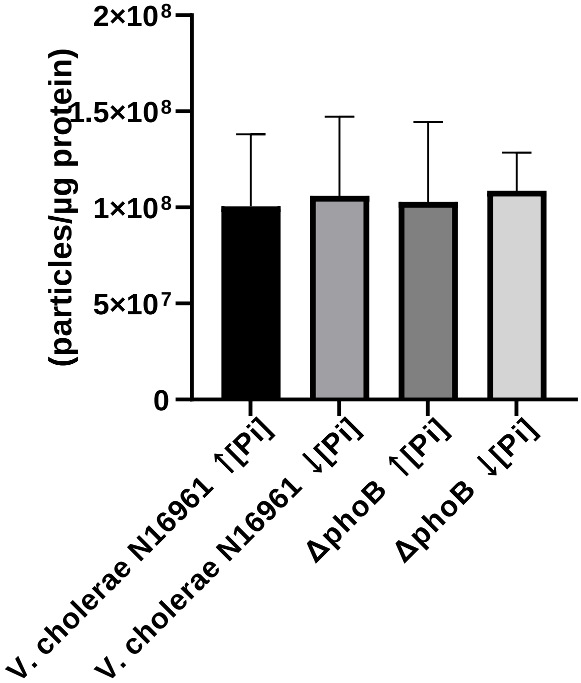

Supplementary Figure S1. Particle-to-protein ratio of OMV preparations from *Vibrio cholerae* N16961 and ΔphoB strains under High and Low phosphate conditions.The ratio between vesicle number and total quantified OMV-associated protein content was calculated for OMV preparations obtained from *V. cholerae* N16961 and ΔphoB strains grown under High Pi and Low Pi conditions. Bars indicate mean ± SD.
